# Malnutrition and Frailty Are Associated with a Higher Risk of Prolonged Hospitalization and Mortality in Hospitalized Older Adults

**DOI:** 10.3390/nu17020221

**Published:** 2025-01-08

**Authors:** Hsiang-Kuang Tseng, Yun-Ju Cheng, Hui-Kung Yu, Kuan-Ting Chou, Chin-Yen Pang, Gwo-Chi Hu

**Affiliations:** 1 Division of Geriatric Medicine, Department of Internal Medicine, MacKay Memorial Hospital, Taipei 104217, Taiwan; eric120008@gmail.com; 2 Department of Medicine, MacKay Medical College, New Taipei City 252005, Taiwan; 3 Department of Long-Term Care, College of Nursing, Asia University, Taichung 413305, Taiwan; dabby630311@gmail.com; 4 Department of Nursing, Mackay Junior College of Medicine, Nursing, and Management, Taipei 112021, Taiwan; hui.kung.yu@gmail.com; 5 Department of Rehabilitation Medicine, Mackay Memorial Hospital, Taipei 104217, Taiwan; j92185@gmail.com (K.-T.C.); ian61015@gmail.com (C.-Y.P.)

**Keywords:** frailty, hospitalization, length of stay, malnutrition, mortality

## Abstract

**Background/Objectives:** Malnutrition and frailty are independent risk factors of prolonged hospitalization and mortality, respectively. However, the combined association of these conditions with the risk of prolonged hospitalization and mortality in hospitalized elderly patients remains unclear. Our object was to investigate the combined association of malnutrition and frailty on the risk of prolonged hospitalization and mortality in hospitalized elderly patients. **Methods:** The current study was a retrospective analysis of 470 patients admitted to the geriatric care unit of a tertiary hospital in Taiwan between 1 August 2019 and 31 March 2023. The Mini Nutritional Assessment-short form and Clinical Frailty Scale were used as evaluation tools for nutritional and frailty status, respectively. Patients were divided into four groups based on nutritional and frailty status. The association between these conditions and the risk of prolonged hospitalization and mortality was investigated using multivariate logistic and Cox proportional hazard models and adjusting for potential confounders. **Results:** Among 470 patients, 144 (31%) exhibited no malnutrition risk or frailty, 146 (31%) exhibited malnutrition risk but no frailty, 46 (10%) exhibited frailty but no malnutrition risk, and 134 (28%) exhibited both malnutrition risk and frailty. Compared to patients with neither condition, those with both conditions had higher risks of prolonged hospitalization (odds ratio 3.23, 95% confidence interval [CI] 1.68–6.12) and mortality (hazard ratio 4.33; 95% CI 2.01–9.34). **Conclusions:** The co-occurrence of malnutrition and frailty has significant detrimental impacts on the risk of prolonged hospitalization and mortality in hospitalized older adults. The findings of this study emphasize the importance of early screening and intervention for malnutrition and frailty among hospitalized elderly patients.

## 1. Introduction

Malnutrition is common in older adults upon hospital admission. Many studies have shown that malnutrition has severe implications for recovery from disease, surgery, or trauma, worsening the prognosis and increasing susceptibility to infections and healthcare costs [1,2]. Therefore, malnutrition is associated with a higher risk of in-hospital morbidity and mortality, in addition to a higher mortality rate in the short and long term after discharge [3].

Frailty refers to a diminished reserve capacity in the major organ systems, and like malnutrition, it is common in hospitalized populations. Frail older adults are highly vulnerable to adverse health outcomes when exposed to physical stress, with hospitalization being one of the most stressful events. Complications of frailty in hospitalized older adults include increased length of hospital stay, higher readmission rates, and a higher risk of mortality [4].

Although they are distinct conditions, malnutrition and frailty share many pathophysiological pathways, including the loss of body tissue and a low inflammatory state [5,6]. Both syndromes have similar etiological factors, such as reduced food intake, hormonal changes, increased energy requirements, and reduced physical activity [7,8]. As a result, a significant proportion of hospitalized older adults are likely to have malnutrition and frailty [9,10]. A recent systematic review and meta-analysis of 10 studies including 2427 hospitalized older adults [11] revealed a strong association and considerable overlap (49.7%) between malnutrition and frailty. Co-occurring frailty and malnutrition may have cumulative effects on health-related outcomes. Most previous studies have focused on malnutrition or frailty alone; few studies have evaluated the impact of their co-occurrence in the same population, which can have a more significant effect, especially in hospitalized older adults.

Therefore, this study aimed to investigate the combined effects of malnutrition and frailty on in-hospital outcomes and mortality among hospitalized older adults. We hypothesized that co-occurring malnutrition and frailty would be associated with a greater risk of poor in-hospital outcomes and mortality than the presence of each condition alone.

## 2. Materials and Methods

We retrospectively reviewed the medical records and database and extracted relevant data from consecutive older patients admitted to the geriatric care unit (GCU) of a tertiary hospital in Taiwan between 1 August 2019 and 31 March 2023. GCUs mainly provide medical care to elderly patients hospitalized for internal diseases. Patients with incomplete nutritional and frailty assessments were excluded. The Institutional Review Board of the Mackay Memorial Hospital reviewed and approved the study protocol (No.23MMHIS184e, approved on 27 June 2023).

Details regarding clinical history, demographic characteristics, laboratory results, and geriatric assessment findings were extracted from the medical records and a registry database. Nutritional status was assessed using the Short-form Mini Nutritional Assessment (MNA-SF), a validated nutritional risk screening tool developed for hospitalized older adults. The MNA-SF includes the following six items: body mass index (BMI), recent weight loss, appetite or eating problems, mobility impairment, acute illness/psychological stress, dementia, and depression, with scores ranging from 0 to 14 points. Patients were classified as normal if their score was ≥12 points, at nutritional risk if their score was 8–11 points, and malnourished if their score was ≤7 points. For the current study, those who were at nutritional risk and malnourished were grouped into the “malnutrition risk” category, as both are considered risk factors within the older adult population. The remaining category was “normal nutrition” [12].

Frailty was assessed using the Clinical Frailty Scale (CFS), a simple and valid tool for defining and grading frailty based on clinical descriptors from routine assessments. The CFS ranges from very fit (score 1) to well (score 2), managing well (score 3), vulnerable (score 4), mildly frail (score 5), moderately frail (score 6), severely frail (score 7), very severely frail (score 8), and terminally ill (score 9) [13]. In the current study, participants were considered frail if they scored ≥5 [14]. At our institution, trained nurses routinely rate the MNA-SF and CFS scores of all patients upon admission.

Cognitive status was assessed using a short portable mental status questionnaire (SPMSQ). This widely employed cognitive assessment tool has proven to be a sensitive and specific screening test for cognitive impairment [15]. Based on previous studies, a three-error cutoff was used to define cognitive impairment [16]. The risk of depressive syndrome was assessed using the 15-item Geriatric Depression Scale (GDS-15). The total GDS-15 score ranges from 0 to 15, and a score of  ≥6 is defined as the presence of depressive symptoms [17].

The demographic characteristics of interest included age, sex, and BMI. The comorbidities included diabetes mellitus, hypertension, atrial fibrillation, coronary artery disease, stroke, and hyperlipidemia. Fasting blood samples from routine hospital admission laboratories were analyzed.

The assessed outcomes included length of hospitalization and all-cause mortality, which were obtained from a clinical database or telephone contact by trained staff. The length of hospitalization was estimated as days from the date of admission to discharge. Prolonged hospitalization was considered ≥11 days, according to the patients’ median length of stay. The index date was defined as the date of admission. Follow-up was started at the index date and ended on 31 December 2023. Survival time was calculated from the index date to the date of death. Patients still alive on 31 December 2023 were censored.

### Statistical Analysis

Descriptive statistics were used to compare baseline characteristics and clinical variables between the patient groupings by nutritional and frail status. Student’s *t*-test and the chi-squared test were applied to compare statistical differences among groups.

The associations of malnutrition and frailty with the risk of prolonged hospitalization and mortality were assessed using multivariate logistic regression models and Cox proportional hazard models to estimate odds ratios (ORs) and hazard ratios (HRs) and 95% confidence intervals (CIs), respectively.

To assess the combined effect of malnutrition risk and frailty on prolonged hospitalization and mortality, we classified the patients according to malnutrition risk and frailty status, yielding four groups: normal, malnutrition risk only, frailty only, and the co-occurrence of malnutrition risk and frailty. Kaplan–Meier curves were plotted according to four-level combined malnutrition and frailty groups, and log-rank tests were applied to compare the survival distributions among the different groups. We also explored the combined effect of malnutrition risk and frailty on the risk of prolonged hospitalization and mortality. Estimated ORs and HRs were adjusted for the following model covariates: age, sex, body mass index, diabetes, coronary artery disease, history of stroke, depressive syndrome, cognitive impairment, and laboratory values. A univariate analysis was followed by a multivariate analysis using the forward method. Variables with statistical significance in the univariate analysis were included in the multivariate analysis. All tests were two-tailed, and a *p*-value of <0.05 was considered statistically significant. All statistical analyses were performed using SAS, version 9.0 (SAS Institute, Cary, NC, USA).

## 3. Results

Data were collected from 470 hospitalized older patients who met the inclusion criteria, including 203 men (43%) and 267 (57%) women, with a mean age of 80.6 ± 7.7 years (range 65–98 years). The median CFS score was 4 (range 0–9), and 180 (38%) subjects had CFS scores ≥ 5. Based on MNA-SF scores, 190 (40%) subjects exhibited normal nutrition (well-nourished), and 280 (60%) were at risk/malnourished. Of the 470 patients, 144 patients (31%) were without malnutrition risk or frailty, 146 (31%) had malnutrition risk but no frailty, 46 (10%) had frailty but no malnutrition risk, and 134 (28%) had both malnutrition risk and frailty. Table 1 presents the baseline demographic and clinical characteristics among different groups. Of patients with malnutrition risk, those who also exhibited frailty were older and more likely to have cognitive impairment, dyslipidemia, and lower BMIs and hemoglobin and albumin levels compared to those without frailty. Of patients with normal nutrition, those who exhibited frailty were older and more likely to have cognitive impairment, atrial fibrillation, and lower albumin levels than those without frailty.

Table 2 displays the logistic regression models evaluating the independent associations of malnutrition risk and frailty on prolonged hospitalization. After adjusting for potential covariates, malnutrition risk was associated with a 54% increased risk of prolonged hospitalization (OR 1.54, 95% CI 1.01–2.36), whereas frailty was also significantly associated with a higher risk of prolonged hospitalization (OR 2.04, 95% CI 1.31–3.16).

Table 3 shows the Cox proportional hazards model evaluating the independent associations of malnutritional risk and frailty on all-cause mortality. For both the unadjusted and adjusted models, malnutrition risk and frailty were independently associated with increased all-cause mortality risk. After adjustment, malnutrition risk was associated with an approximately twofold risk of mortality (HR 2.05, 95% CI 1.16–3.63), and frailty was also associated with a higher risk of mortality (HR 2.02, 95% CI 1.27–3.20).

**Figure 1:**
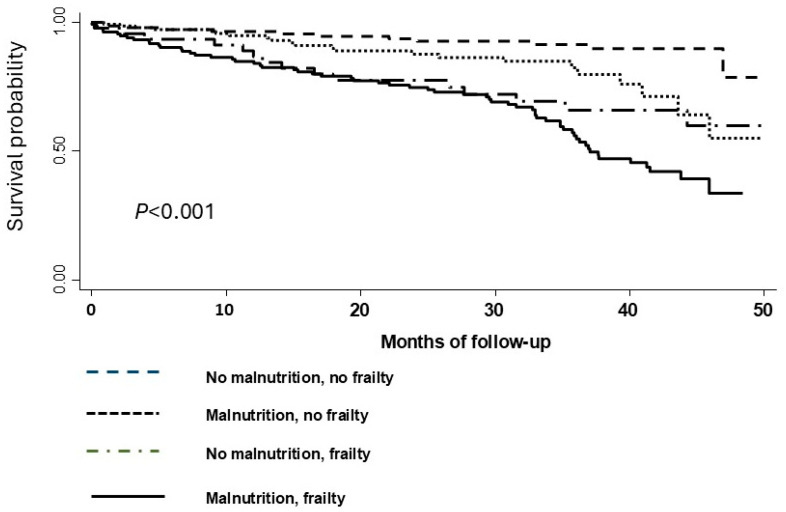
Kaplan–Meier curves were depicted by four-level groups according to malnutrition and frailty status.

During the median 27.1-month observational period, there were 71 (53%) deaths in patients with malnutrition risk and frailty, 14 (30%) in those with frailty but without malnutrition risk, 22 (15%) in those with malnutrition risk and without frailty, and 12 (8%) in the reference group (patients with neither condition). Figure 1 illustrates the Kaplan–Meier survival curves for four-level groups according to malnutrition risk and frail status at admission, revealing significant differences in survival patterns (log-rank test, *p* < 0.001).

The combined associations of malnutrition risk and frailty categories on the risk of prolonged hospitalization and mortality are shown in Table 4. Patients with both malnutrition risk and frailty had the highest risks of prolonged hospitalization (OR 3.23, 95% CI 1.68–6.12) and mortality (HR 4.33, 95% CI 2.01–9.34) compared to patients with neither condition. Patients with frailty but without malnutrition risk also had higher risks of prolonged hospitalization (OR 1.65, 95% CI 0.77–3.56) and mortality (HR 2.87, 95% CI 1.23–6.71) than patients with neither condition, as did those with malnutrition risk but without frailty (OR 1.11, 95% CI 0.62–2.01 for prolonged hospitalization; HR 1.86, 95% CI 0.86–4.06 for mortality).

## 4. Discussion

Our study showed that the malnutrition risk defined by the MNA-SF was significantly associated with adverse outcomes, including a higher risk of prolonged hospitalization and mortality in older patients hospitalized in acute GCUs. Similarly, frail patients had a significantly higher risk of prolonged hospitalization and mortality than non-frail hospitalized older adults. A novel finding of our study was that older hospitalized adults with co-occurring malnutrition and frailty had a significantly higher risk of adverse outcomes and mortality. Notably, patients experiencing both conditions concurrently have the highest risk of adverse outcomes, underscoring the importance of jointly considering these factors in geriatric risk assessments.

Malnutrition in hospitalized patients may impair many physiological functions, such as impairing the immune system, delaying wound healing, and reducing the response to medical treatment. Some studies have found that malnutrition is associated with a high risk of prolonged hospitalization and mortality in different clinical patients, including medical, surgical, trauma, and cancer patients [3,18,19]. Serum albumin levels are commonly used as clinical markers in the assessment of the nutritional status among hospitalized older adults. Hypoalbuminemia is the result of the combined effects of nutritional deterioration and disease-related inflammatory response. Hypoalbuminemia has been identified as a significant prognostic factor across various clinical settings, indicating its association with adverse outcomes in different patient populations [20,21]. Our study confirmed the results of these investigations using different malnutrition screening tools and clinical markers in hospitalized older adults. Several studies have investigated the role of frailty in increasing morbidity and mortality among hospitalized older adults. In a systematic review and meta-analysis, Cunha et al. showed that frailty is a risk factor for adverse outcomes, such as extended hospital stays and mortality, in hospitalized older adults [4]. Similarly, we reaffirmed the significant association between frailty and a higher risk of prolonged hospitalization and mortality in hospitalized older adults.

This study’s novelty lies in assessing coexisting malnutrition and frailty in hospitalized older adults. Patients with a co-occurrence of malnutrition and frailty tended to have a significantly higher risk of prolonged hospitalization and mortality than those without this combination. Some studies have investigated the combined effect of malnutrition and frailty on clinical outcomes in patients undergoing hip surgery or hospitalized for acute decompensated heart failure, with similar findings reported [22,23,24,25]. There are several possible explanations for our results. Malnutrition and frailty are related to several biological changes, such as increased inflammation and oxidative stress, immune activation, and mitochondrial dysfunction [26,27]. The co-occurrence of malnutrition and frailty in hospitalized older patients might be an expression of such biological alterations [28], thus indicating an interaction. Increased inflammation and oxidative stress are involved in several pathologies and are recognized as a pivotal factor that contributes to disease-related complications and mortality [29]. Recent evidence indicates that hypo-perfused patients without congestion profile-type heart failure commonly present with malnutrition, severe frailty, and neurological symptoms. This heart failure phenotype is associated with poor prognosis over short-term follow-up periods [30]. Secondly, malnutrition is a complex syndrome encompassing an imbalance between nutrient intake and uptake and the patient’s metabolic needs, which can be further aggravated in the presence of a disease-related inflammatory response [31]. Malnutrition in hospitalized patients is associated with adverse clinical outcomes, including the loss of muscle mass and strength, diminished physical function, and impaired frailty [32]. Additionally, frailty is associated with inflammatory responses and oxidative stress. Oxidative stress reactions and inflammation can lead to anorexia, reduced food intake, and catabolic states, which can worsen the nutritional status [33].

The present study had some limitations. First, this study was retrospective in design and potentially prone to systematic bias; however, this design may make this study more likely to reflect actual clinical practice, avoiding the danger of the data collection process influencing clinical care decisions or introducing bias into the study recruitment process. Second, the MNA-SF is a validated instrument for screening malnutrition in geriatric hospital patients; however, no comprehensive nutritional assessment is available to confirm the diagnosis of malnutrition. Similarly, in the case of frailty assessment, the CFS is a relatively easy tool to implement; it has some subjective components that can lead to interobserver variability. Third, the MNA-SF and CFS are continuous variables that are transformed into categorical variables when combined. Therefore, further clarification is needed to determine the optimal cutoff values for the MNA-SF and CFS. Fourth, the specific causes of mortality were not available; thus, we could not provide data regarding the association of specific causes of mortality, such as cardiovascular death, which has been reported to be associated with frailty or malnutrition. Finally, although multivariate analysis was used to adjust for potential confounding factors, residual unmeasured confounding factors may remain.

Our results highlighted that older hospitalized adults with co-occurring malnutrition and frailty had a significantly higher risk of adverse outcomes. The results of this study suggest that early malnutrition and frailty screening are essential aspects of comprehensive care in patients in GCUs as they might help to predict poor clinical outcomes and, thus, indirectly predict post-discharge mortality [34]. Our findings also suggest that a substantial proportion of hospitalized older adults have coexisting malnutrition and frailty; therefore, additional support may be required to treat these patients.

## 5. Conclusions

Our results highlighted the high prevalence of co-occurrence of malnutrition and frailty among hospitalized older adults. This combination significantly impacted the risks of prolonged hospitalization and mortality. Further research is necessary to better understand these complex interactions and evaluate the effectiveness of intervention programs to prevent or treat the onset of malnutrition and frailty during hospitalization.

## Figures and Tables

**Table 1:** Baseline demographic and clinical characteristics of patients according to nutritional and frailty status.

	Malnutrition Risk N, 280		*p* Value	Normal Nutrition N, 190		*p* Value
Parameters	Frailty N, 134	Non-Frailty N, 146		Frailty	Non-Frailty	
Age (years)	83.2 ± 7.3	79.3 ± 7.4	<0.001	83.5 ± 7.7	78.7 ± 7.5	<0.001
BMI (kg/m^2^)	21.8 ± 4.8	23.0 ± 3.8	0.002	26.6 ± 6.4	25.8 ± 3.6	0.29
Female	88 (66%)	86 (59%)	0.24	25 (54%)	68 (47%)	0.4
Cognitive impairment	101 (75%)	61 (41%)	0.001	35 (76%)	64 (44%)	<0.001
Depression syndrome	72 (53%)	64 (43%)	0.09	16 (35%)	39 (27%)	0.3
Diabetes mellitus	52 (38%)	50 (34%)	0.4	25 (54%)	71 (49%)	0.55
Hypertension	83 (62%)	93 (63%)	0.76	32 (69%)	106 (73%)	0.59
CAD	19 (14%)	26 (17%)	0.4	9 (19%)	43 (30%)	0.17
stroke	21 (16%)	68 (47%)	0.4	9 (19%)	18 (13%)	0.23
Atrial fibrillation	27 (20%)	26 (18%)	0.63	12 (26%)	16 (11%)	0.01
Dyslipidemia	36 (27%)	72 (49%)	<0.001	25 (54%)	68 (47%)	0.4
Blood glucose (mg/dL)	134.7 ± 97.1	118.7 ± 39.3	0.07	126.5 ± 55.6	127.8 ± 45.2	0.88
triglyceride (mg/dL)	106.8 ± 63.1	108.1 ± 54.9	0.85	112.6 ± 61.3	116.6 ± 64.3	0.71
Hemoglobin (g/dL)	10.9 ± 1.9	11.8 ± 1.9	<0.001	11.7 ± 2.4	12.2 ± 1.8	0.14
Creatinine (mg/dL)	1.3 ± 0.9	1.2 ± 0.8	0.3	1.5 ± 0.9	1.3 ± 0.9	0.17
Uric acid (mg/dL)	5.8 ± 2.5	5.8 ± 1.8	0.96	6.3 ± 1.8	5.9 ± 1.7	0.17
Albumin (mg/dL)	3.4 ± 0.6	3.6 ± 0.7	<0.001	3.8 ± 0.6	4.0 ± 0.6	0.02

Abbreviations: BMI, body mass index; CAD, coronary artery disease. Data are presented as mean ± SD or number (%).

**Table 2:** The logistic regression model evaluates the independent associations of malnutrition risk and frailty on prolonged hospitalization.

	Univariable Analysis		Multivariable Analysis	
Variables	OR, 95%CI	*p* Value	OR, 95%CI	*p* Value
Malnutrition risk	1.89 (1.27–2.80)	<0.001	1.54 (1.01–2.36)	0.04
Frailty	2.65 (1.80–3.91)	<0.001	2.04 (1.31–3.16)	0.002
Covariates included in the adjustment				
Age, years	1.01 (0.98–1.03)	0.71		
Body mass index	0.98 (0.95–1.03)	0.56		
Male	1.03 (0.71–1.51)	0.87		
Cognitive impairment	1.47 (1.01–2.16)	0.04	1.04 (0.68–1.58)	0.84
Depression syndrome	1.85 (1.26–2.70)	0.002	1.61 (1.08–2.42)	0.02
Diabetes mellitus	1.23 (0.83–1.80)	0.27		
Hypertension	1.03 (0.69–1.54)	0.87		
Coronary artery disease	0.80 (0.50–1.28)	0.35		
Stroke	1.04 (0.62–1.74)	0.87		
Atrial fibrillation	0.78 (0.46–1.33)	0.37		
Dyslipidemia	0.85 (0.58–1.24)	0.39		
Glucose	1.00 (0.99–1.01)	0.13		
Triglyceride	1.00 (0.99–1.01)	0.95		
Hemoglobin	0.84 (0.76–0.93)	<0.001	0.91 (0.82–1.02)	0.09
Creatinine	1.29 (1.04–1.61)	0.02	1.22 (0.96–1.53)	0.13
Uric acid	1.07 (0.97–1.18)	0.15		
Albumin	0.75 (0.56–0.99)	0.04	0.98 (0.71–1.33)	0.92

Abbreviations, OR, odds ratio; CI, confidence interval.

**Table 3:** The Cox proportional hazards model evaluates the independent associations of malnutrition risk and frailty on all-cause mortality.

	Univariable Analysis		Multivariable Analysis	
Variables	HR, 95%CI	*p* Value	HR, 95%CI	*p* Value
Malnutrition risk	3.10 (2.00–4.79)	<0.001	2.05 (1.16–3.63)	0.01
Frailty	3.67 (2.46–5.46)	<0.001	2.02 (1.27–3.20)	0.003
Covariates included in the adjustment				
Age, years	1.01 (0.98–1.03)	<0.001	1.10 (1.06–1.13)	<0.001
Body mass index	0.98 (0.95–1.03)	<0.001	0.93 (0.88–0.98)	<0.001
Male	1.03 (0.71–1.51)	0.22		
Cognitive impairment	1.47 (1.01–2.16)	0.75		
Depression syndrome	1.85 (1.26–2.70)	0.88		
Diabetes mellitus	1.23 (0.83–1.80)	0.56		
Hypertension	1.03 (0.69–1.54)	0.09		
Coronary artery disease	0.80 (0.50–1.28)	0.96		
Stroke	1.04 (0.62–1.74)	0.31		
Atrial fibrillation	0.78 (0.46–1.33)	0.01	1.16 (0.70–1.93)	0.55
Dyslipidemia	0.85 (0.58–1.24)	0.17		
Glucose	1.00 (0.99–1.01)	<0.001	0.99 (0.99–1.02)	0.88
Triglyceride	1.00 (0.99–1.01)	0.06		
Hemoglobin	0.84 (0.76–0.93)	<0.001	0.87 (0.77–0.99)	0.03
Creatinine	1.29 (1.04–1.61)	0.37		
Uric acid	1.07 (0.97–1.18)	0.07		
Albumin	0.75 (0.56–0.99)	<0.001	0.62 (0.44–0.87)	0.005

Abbreviations, HR, hazard ratio; CI, confidence interval.

**Table 4:** Univariable and multivariable models evaluating the combined associations of malnutrition risk and frailty on the risk of prolonged hospitalization and all-cause mortality.

	Univariable Analysis		Multivariable Analysis	
Prolonged Hospitalization	OR, 95%CI	*p* Value	OR, 95%CI *	*p* Value
No malnutrition, no frailty	Reference		Reference	
Malnutrition, no frailty	1.29 (0.77–2.15)	0.33	1.11 (0.62–2.01)	0.72
No malnutrition, frailty	1.70 (0.84–3.43)	0.14	1.65 (0.77–3.56)	0.20
Malnutrition, frailty	3.68 (2.21–6.10)	<0.001	3.23 (1.68–6.12)	<0.001
Mortality	HR (95%CI)	*p* value	HR (95%CI) **	*p* value
No malnutrition, no frailty	Reference		Reference	
Malnutrition, no frailty	2.37 (1.17–4.80)	0.02	1.86 (0.86–4.06)	0.11
No malnutrition, frailty	3.22 (1.48–6.97)	0.003	2.87 (1.23–6.71)	0.01
Malnutrition, frailty	7.05 (3.82–13.00)	<0.001	4.33 (2.01–9.34)	<0.001

Abbreviations, OR, odds ratio; CI, confidence interval; HR, hazard ratio. * Adjusted by cognitive impairment, depression syndrome, hemoglobin, creatinine, and albumin level. ** Adjusted by age, body mass index, atrial fibrillation, glucose, hemoglobin, and albumin level.

## Data Availability

The datasets generated and analyzed during the current study are not publicly available but are available from the corresponding author upon reasonable request.
